# Risk Evaluation Requires an Independent Mind

**DOI:** 10.3390/diseases5040028

**Published:** 2017-11-24

**Authors:** Christian Schmidt, Joachim Storsberg

**Affiliations:** Fraunhofer Institute Applied Polymer Research (IAP), Division of Life Science and Bioprocesses, Department of Biomaterials and Healthcare, Potsdam-Golm 14476, Germany; joachim.storsberg@iap.fraunhofer.de

**Keywords:** Risk assessment, bias, research

## Abstract

Biomedical research pertaining to pathologies observed in adolescents can involve areas where patients can expect no immediate benefits. Here, Congress stipulates that local review boards are restricted to approving procedures posing no greater than minimal risk (45 CFR 46.404). An evaluation of risk embraces the current state of the art with regard to the safety and efficacy of procedures. A tendency of biomedical scholars to cite highly cited documents can introduce a bias in an argumentation in favor or against a given recommendation in the context that bias in citations can be correlated with an imprudent use of funds for research. We use choice examples to highlight the necessity of approaching any scholarly task with an independent mind.

Progress in biomedical sciences is measured by comparing new publications against the existing precedent. The inherent problem with this comparison is that what we term existing precedent is, by and of itself, not absolute in the sense that it is dependent on the context of its creation, or, in other words, as good as the understanding of a given problem was at the time of the writing and revision of the respective manuscript. Because our understanding of the intricacies of biological systems continuously progresses, using an, phrased in the extreme, obsolete standard renders the progress of life sciences accessible only in the context of contemporary papers and references therein. Where can one turn since we, to the best of our knowledge, are forced to concede that assessing the knowledge extracted from literature in biosciences is only valid if truly contemporary references are used? How far can one stretch the credible meaning of ‘contemporary references’ with the apparent preference of biomedical scholars to cite highly cited papers [1,2]? This bias could lead to unintended consequences, such as “sins of omission” with, in extreme cases, consequences relating to the validity of results [3,4], up to what Congress termed "minimal risk" as a guidance for review boards as they consider the approval of procedures [5].

If the information we assume that we have, or the perception of whatever we think, is the real and complete information about the given system we are investigating, whatever it is that causes the quantum entanglement to collapse [6] could also result in a subset of realizations of probability functions that mirrors the limitation we introduce before we start planning or conducting the experiment. This phenomenon, seen in the realm of quantum entanglement, may not be so very different from deciphering what we seem to label ‘background noise’ in life sciences [7]. Are we, in other words, limiting the truly possible outcomes of an assay by our prejudice regarding the experiment, the model chosen, and the analytics employed to acquire quantifiable data from this very assay? If so, what truly independent reference is appropriate to avoid such bias?

Because of the inherent limitation discussed above, one could perhaps turn to so far undisputed lessons drawn from other areas of scholar activity, or, equally as important, universally applicable guidance that could be useful in addressing the problem of bias that, by, of, and in of itself, constitutes an inherent limitation of the validity of the observation of an experimental model.

In law, what is termed an independent mind of a person not vested in the outcome of a given case, or resolution of a dispute, has been a time-honored approach to ascertain the weight and quality of the adversarial presentation of aspects or viewpoints of a given issue, assuming that the arguments or viewpoints presented fully encompass the aspects of the problem, which shall be assumed charitably for the sake of this argument [8]. This, however, still does not adequately address a true point of reference to what we have termed ‘independent mind’. Perhaps a resolution of this conundrum can be found in means of expressions that do not involve the inherent imprecision of language [9], say, music and arts.

Music and arts can be likened as a medium to convey sentiments throughout the ages, which can certainly inspire the mind of the beholder or listener, regardless of the state of mind [10]. On occasion, focus on the art itself for the sake of focusing on the art can provide a vehicle to sort mental processes to yield what is described as focusing on the current mental task or problem to be pondered [11]. What is it that music and art provide that so many scholars use to purge one’s mind? Could it be an alternate form of expression, which conveys ideas and concepts without words, in perfect harmony with the means of the expression of ideas and concepts using words [12]?

Embracing these independent strains of communication, and other means, could be summarized as a complete expression of what we perceive our surrounding environment to be with our senses and methods of logical deduction. Calming one’s senses by removing one’s ego from processing intellectual problems via aligning the mind and ideals with concepts, validated throughout the ages, can serve us well in conserving intellectual energy as each of us ponders seemingly intractable intellectual assignments in an increasingly complex scientific endeavor [13]. 
